# Genome-wide association study of genetic markers of coat color patterns in Sumatran native cattle

**DOI:** 10.14202/vetworld.2024.2537-2543

**Published:** 2024-11-13

**Authors:** Hartati Hartati, Widya Pintaka Bayu Putra, Eko Handiwirawan, Erpan Ramon, Jhon Firison, Zubir Zubir, Nandari Dyah Suretno, Mariyono Mariyono, Yenni Yusriani, Dewi Khosiya Robba, Alfian Destomo, Tika Anggraeni, Pajri Anwar, Sulistiyoningtiyas Irmawanti, Aprisal Aprisal, Simon Elieser, Dian Kurniawati

**Affiliations:** 1 Research Center for Animal Husbandry, National Research and Innovation Agency (BRIN), Bogor 16911, Indonesia; 2 Research Center for Applied Zoology, National Research and Innovation Agency (BRIN), Bogor 16911, Indonesia; 3 Research Center for Sustainable Production System and Life Cycle Assessment, National Research and Innovation Agency (BRIN), Banten, Indonesia; 4 Directorate of Laboratory Management, Research Facilities, and Science and Technology Areas, Deputy for Research and Innovation Infrastructure, National Research and Innovation Agency (BRIN), Indonesia; 5 Department of Animal Science, Faculty of Agriculture, Islamic University of Kuantan Singingi, Teluk Kuantan Riau, Indonesia; 6 Department of Animal Husbandry and Veterinary Science, West Sumatra Provincial Government, Rasuna Said Street No. 68 Padang, Indonesia; 7 Department of Animal Science, Faculty of Agriculture, University of Lampung 35145, Indonesia

**Keywords:** cattle, coat color, functional gene, genome-wide association study, Sumatra

## Abstract

**Background and Aim:**

Coat color is a phenotypic trait that is affected by many functional genes. In addition, coat color is an important characteristic of breeds in livestock. This study aimed to determine functional genes for coat color patterns in Sumatran native cattle in Indonesia using a genome-wide association study method.

**Materials and Methods:**

A bovine single nucleotide polymorphism (SNP) 50K BeadChip was used for the investigation. A total of 46. Sumatran native cattle of three colors as follows: Brown (36 animals), white (9 animals), and black (1 animal), were used as experimental animals.

**Results:**

Results showed that the SNP markers ARS-BFGL-NGS-75486 (p = 2.46 × 10^-7^) and BTB-01992588 (p = 1.06 × 10^-5^) were selected as two genetic markers for coat color variation in animals under study, which were located at the cytoplasmic FMR1-interacting protein 2 (*CYFIP*2) gene at BTA7 and small G protein signaling modulator 1(*SGSM1*) genes at BTA17, respectively. The polymorphic informative content values of both SNP markers were 0.33 (ARS-BFGL-NGS-75486) and 0.13 (BTB-01992588). In this study, a genetic marker for coat color patterns in Sumatran native cattle was obtained based on the haplotypes of both SNP markers.

**Conclusion:**

It can be concluded that *CYFIP2* and *SGSM1* are two coloration genes that affect the phenotype characteristics of Sumatran native cattle.

## Introduction

Genetic diversity in domestic livestock plays a crucial role in animal management and breeding, particularly in the context of desired phenotypic characteristics. One significant aspect of these phenotypic characteristics is coat color pattern, which not only influences esthetics but also has the potential to be related to other economically or biologically relevant traits and become a distinctive characteristic of a breed. Sumatran native cattle, as a distinctive breed of cattle from Indonesia, have different physical and phenotypic characteristics, including coat color patterns [1–3], making them an interesting subject for genetic studies.

Sumatran cattle are generally maintained for meat production through an extensive management system. At present, several native Sumatran cattle have been designated as animal genetic resources for Indonesian cattle by the Indonesian Ministry of Agriculture [4], including Aceh cattle in Nanggroe Aceh Darussalam Province, Pesisir cattle in West Sumatra Province, Kuantan cattle in Riau Province, and Krui cattle in Lampung Province. However, there are still native cattle in other provinces of Sumatra, such as Jambi and Bengkulu Provinces (Kaur cattle), which are Indonesian cattle germplasm that have the potential to be used for meat production purposes [5–7]. Coat color is an essential trait for breed standardization in livestock animals. Hence, detecting genetic markers that indicate desirable coat color traits is important for determining the standard breed of livestock. Several genes and their corresponding alleles control coat color in cattle, such as *ASIP* (agouti), *TYR* (albinism), *TYRP1* (brown), *KIT* (color sided and dominant white), *KITLG* (roan), *PMEL* (dilution), *MC1R* (extension), and *MITF* (white spotting), as described by Brenig *et al*. [8]. However, a study by Jakaria *et al*. [9] using *MC1R* (exon 1) and *KIT* (exon 3) did not obtain the genetic markers for coat color abnormalities in Bali cattle. Nonetheless, a mutation site in exon 1 of the *TYR* gene (g.939A>G) can be used as a genetic marker for the abnormal coat color of Bali cattle [10]. In addition, mutation sites in exon 3 of *MITF* (c.328C>T) and exon 3 of *ASIP* (c.292C>T) are crucial genes for the white-spotted coat in swamp buffaloes [11] and coat color patterns in Murrah buffaloes [12]. Despite this, two mutation sites of c.649C>T (exon 4) and g.1179+2T>A (intron 9) in the *MITF* gene caused the white-spotted coat in swamp buffaloes [13].

At present, many significant coloration genes in cattle are detected through the genome-wide association study (GWAS) technique. *MITF*, *MC1R, ASIP, KIT*, and *TYR* are known as common coloration genes in cattle [14]. According to GWAS, the *MITF* gene is strongly associated with spotting coat patterns in Ethiopian cattle [15] and Brown Swiss cattle [16]. Subsequently, *PAX3* and *PLK2* are crucial genes affecting the white-spotted coat in dairy cattle [17] and the sidedness coat in Cinisara cattle [18], respectively. In addition, *PMEL* is a crucial gene for the coat color patterns of Fleckvieh [19] and Simmental × Holstein [20] cattle. Therefore, Fan *et al*. [21] revealed that *KIT, IGFBP7, PDGFRA, MITF, ING3*, and *WNT16* are crucial genes for pigmentation in Chinese Holstein cattle. BDNF, *FGF18, CACNA2D1*, and *HGF* are crucial genes for the coat color patterns of Vrindavani cattle [22].

Research on coat color patterns in cattle has identified several essential genes that govern this variation, but specific genetic knowledge of Sumatran cattle is limited. Genomic approaches, such as GWAS, offer opportunities to elucidate the association between genetic variations and coat color phenotypes in this breed. This study aimed to detect the genetic markers of coat color patterns in Sumatran native cattle using GWAS methods. The results of this study are important for developing molecular selection for coat color patterns in Sumatran native cattle, which will be important for breed standardization in the future.

## Materials and Methods

### Ethical approval

This study was approved by the Ethics Committee of Animal Care and Use of the National Research and Innovation Agency (Approval number: 150/KE.02/SK/07/2023).

### Study period and location

**Figure-1:**
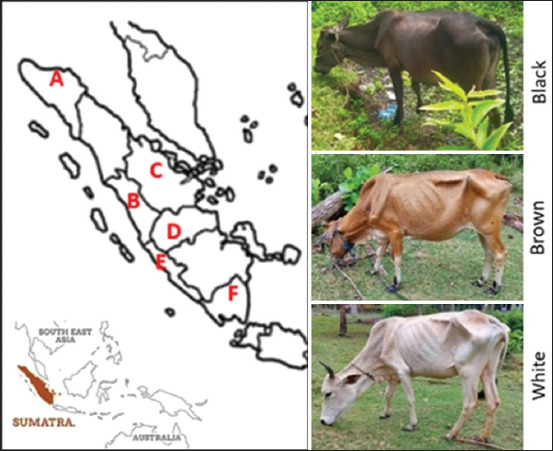
Sampling locations: (A) Aceh, (B) West Sumatra, (C) Riau, (D) Jambi, (E) Bengkulu, and (F) Lampung provinces of Indonesia. Coat color patterns of Sumatran native cattle are described as Black, Brown, and White [Source: https://www.slideshare.net/slideshow/peta-provinsi-indonesia-butapptx/255282074].

The study was conducted from January to June 2024. Sampling was carried out in six pro-vinces on Sumatra island, Indonesia, namely West Sumatra, Riau, Jambi, Bengkulu, Lampung, and the Indrapuri Center for Superior Animal Breeding and Foraged Animal Feed (BPTU-HPT) Nanggroe Aceh Darussalam, Indonesia (Figure-1). DNA analysis was carried out at the Genomic Laboratory, National Research and Innovation Agency, Cibinong, Bogor, Indonesia.

### Animals

A total of 46 cattle (mixed-sex) at an adult age were used as experimental animals. The animals were collected from six provinces on Sumatra island: Aceh (8 heads), West Sumatra (7 heads), Riau (9 heads), Jambi (7 heads), Bengkulu (7 heads), and Lampung (8 heads). In addition, the investigated animals had three coat color patterns: Brown (36 heads), white (9 heads), and black (1 head). Details of the sample locations and coat color patterns of the animals are illustrated in Figure-1. The farmers maintained the animals under an extensive management system.

### DNA extraction and single nucleotide polymorphism (SNP) detection

DNA was extracted from the blood samples of each experimental animal. DNA extraction analysis was performed using a DNA extraction kit (Zymo Research, USA) according to the manufacturer’s instructions. The detection of SNPs in the genomic region of the investigated animals was performed using Bovine SNP50K BeadChip v.3 (Illumina, USA) at the Genomic Laboratory of the National Research and Innovation Agency (BRIN).

### Bioinformatics analysis

#### Filtering and selecting SNP markers

A total of 53,218 SNP markers were obtained from the Bovine SNP50K BeadChip with GenomeStudio*^®^ v.2.0* software (https://www.support.illumina.com). In this study, SNP marker filtering was performed using the TASSEL 5.0 software (https://tassel.bitbucket.io/) [23]. Genomic analysis was performed using autosomal SNP markers. A minor allele frequency of (MAF) <0.05 was not used for analysis. In addition, undetected SNP markers were removed from the genomic analysis. After the filtering analysis, 51,267 SNP markers were selected for genomic analysis in the investigated animals. Subsequently, the best SNP markers were selected based on quantile–quantile (Q–Q) and Manhattan plots. The SNP marker plots above the Bonferroni-corrected threshold line (-Log_10_P^5^) were selected as potential genetic markers for further investigation.

#### Gene mapping

Analysis gene mapping was performed to detect the location of selected SNP markers in the genomic regions of the animals under study. A *Bos taurus* genome reference (Assembly: ARS-UCD2.0/GCF_002263795.3) from the National Center for Biotechnology Information database (https://www.ncbi.nlm.nih.gov) was used to detect the position of the SNP markers. Moreover, a STRING program (https://string-db.org) was used to determine the relationship between the detected genes and other coloration genes.

### Genetic diversity and genetic markers

Genetic diversity analysis was performed to evaluate selected SNP markers associated with genotype and allele frequencies [24], observed and expected heterozygosities [25], polymorphic informative content (PIC), number of effective alleles (n_e_), and Chi-square (χ^2^) values [26]. Subsequently, the genetic markers for coat color patterns were detected by combining the genotype of each SNP marker (haplotyping). Hence, haplotypes found (100%) only in specific coat color groups are described as genetic markers.

## Results

**Figure-2:**
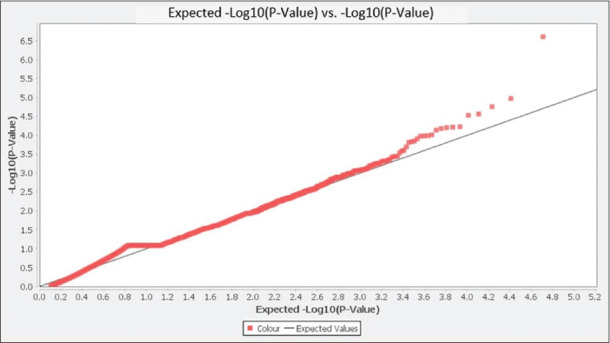
Q–Q plots of the selected p-value (-Log_10_ P^5^) for individual SNP markers (red dots). The threshold line indicates the expected value when confirming the null hypothesis of an absence association. Q–Q=Quantile–quantile, SNP=Single nucleotide polymorphism.

**Figure-3:**
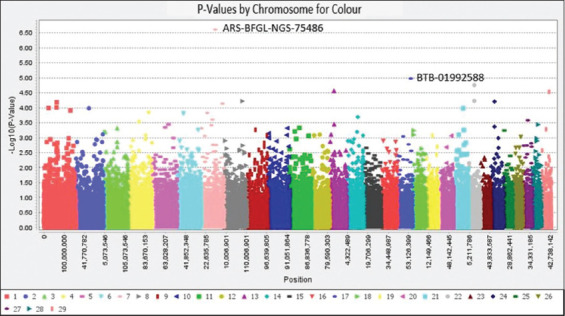
Two selected SNP markers from Manhattan plots of coat color patterns in Sumatran native cattle. The X- and Y-axes show the chromosomal position and -Log_10_ P value, respectively. Colored dots indicate the SNP markers at different chromosomal regions. SNP=Single nucleotide polymorphism.

This study revealed that GWAS could detect genetic markers of coat color patterns in Sumatran native cattle, as shown in the Q–Q plots graphic (Figure-2). According to Q–Q plots, many SNP markers were spread above the Bonferroni-corrected threshold line (-Log_10_P^5^). Hence, it can be suggested that the coat color trait be selected through molecular selection. In addition, two SNP markers of ARS-BFGL-NGS-75486 and BTB-01992588 were detected as the genetic markers for coat color patterns according to the Manhattan plot graphic (Figure-3). The SNP markers of ARS-BFGL-NGS-75486 (p = 2.46 × 10^-7^) and BTB-01992588 (p = 1.06 × 10^-5^) were located at intron 17 of cytoplasmic FMR1-interacting protein 2 (*CYFIP2*) gene at BTA7 and intron 20 of small G protein signaling modulator 1 (*SGSM1*) gene at BTA17, respectively (Table-1).

**Table-1:** Identification of candidate genes for detecting coat color patterns in Sumatran native cattle.

SNP marker	BTA	p-value	Position	MAF	Gene*	Region	Range
ARS-BFGL-NGS-75486	7	2.46×10^-7^	69,004,072	0.31	*CYFIP2*	Intron 17	68,943,185–69,077,507
BTB-01992588	17	1.06×10^-5^	65,071,599	0.07	*SGSM1*	Intron 20	65,010,766–65,084,804

SNP=Single nucleotide polymorphism, BTA=*Bos taurus* autosome, MAF=Minor allele frequency,

* Assembly: ARS-UCD2.0 (GCF_002263795.3)

**Figure-4:**
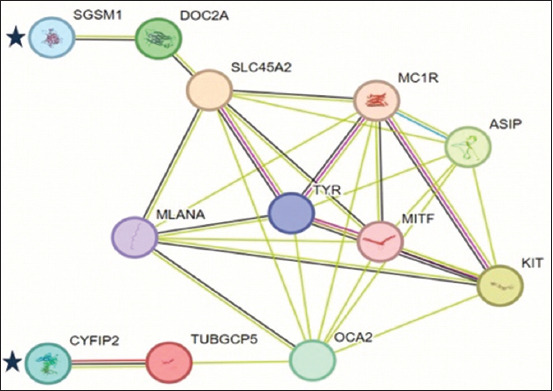
Gene interaction network between detected gene (star symbol) and common coloration genes.

A higher PIC value was obtained using the SNP marker ARS-BFGL-NGS-75486 (0.34), as shown in Table-2. The SNP marker BTB-01992588 had a lower PIC value (0.13). Nonetheless, the genetic diversity of both SNP markers was under genetic equilibrium. Concerning the *CYFIP2* gene (ARS-BFGL-NGS-75486), the AA genotype was observed in black and brown coat cattle. CC and AC genotypes were observed in white and brown coat cattle (Table-3). In the *SGSM1* gene (BTB-01992588), a genotype was observed in white and brown coat cattle. The GG genotype was observed in the brown coat cattle. Thus, the AG genotype is present in black and white coat cattle. However, the AA/AG haplotype was detected in black coat cattle. The white coat cattle had four haplotypes: CC/AA, CC/AG, AC/AA, and AC/AG (Table-4). Therefore, the brown coat cattle had six haplotypes: AA/AA, AA/GG, CC/AA, CC/GG, AC/AA, and AC/GG. Moreover, *CYFIP2* and *SGSM1* genes are indirectly unrelated to the common coloration genes (*TYR, MITF, KIT, MC1R*, and *ASIP*). Therefore, at least five genes (tubulin gamma complex protein *5* [*TUBGCP*5]*, OCA2, MLANA*, solute carrier family 45 member *2* [*SLC45A*2], and double C2-like domain-containing protein alpha [*DOC2*A]) are related to *CYFIP2, SGSM1*, and common coloration genes (Figure-4).

**Table-2:** Genetic diversity in selected SNP markers of coat color patterns in Sumatran native cattle.

SNP marker	Genotype frequency (N)	Allele frequency	H_o_	H_e_	PIC	n_e_	Chi-square
ARS-BFGL-NGS-75486	AA = 0.56 (25)	A = 0.68	0.28	0.43	0.34	1.76	5.49*
	CC = 0.16 (8)	C = 0.32					
	AC = 0.28 (13)						
BTB-01992588	AA = 0.88 (42)	A = 0.92	0.11	0.14	0.13	1.16	2.37*
	GG = 0.02 (1)	G = 0.08					
	AG = 0.10 (5)						

SNP=Single nucleotide polymorphism, N=Number of individuals, H_o_=Observed heterozygosity, H_e_=Expected heterozygosity, PIC=Polymorphic informative content, n_e_=Number of effective allele,

* Under genetic equilibrium

**Table-3:** Genotype distribution of the coat color patterns of Sumatran native cattle.

SNP marker	Gene	Genotype	Percentage (N)		

			Black	Brown	White
		AA	1.00 (1)	63.88 (23)	0.00 (0)
ARS-BFGL-NGS-75486	*CYFIP2*	CC	0.00 (0)	0.03 (1)	88.89 (8)
		AC	0.00 (0)	33.12 (12)	11.11 (1)
		AA	0.00 (0)	97.22 (35)	55.56 (5)
BTB-01992588	*SGSM1*	GG	0.00 (0)	0.03 (1)	0.00 (0)
		AG	1.00 (1)	0.00 (0)	44.44 (4)

N=Number of observations, SNP=Single nucleotide polymorphism, *CYFIP2*=Cytoplasmic FMR1-interacting protein 2, *SGSM1*=Small G protein signaling modulator 1

**Table-4:** Haplotype variation in coat color patterns of Sumatran native cattle.

Coat color	Haplotype (*CYFIP2*/*SGSM1*)
Black	AA/AG
Brown	AA/AA; AA/GG; CC/AA; CC/GG; AC/AA; AC/GG
White	CC/AA; CC/AG; AC/AA; AC/AG

*CYFIP2*=Cytoplasmic FMR1-interacting protein 2, *SGSM1*=Small G protein signaling modulator 1

## Discussion

Two genetic markers in the coat color patterns of Sumatran native cattle were detected using a Bovine SNP50K BeadChip, that is, ARS-BFGL-NGS-75486 (*CYFIP2* gene) and BTB-01992588 (*SGSM1* gene). According to GenBank, SNP markers of ARS-BFGL-NGS-75486 and BTB-01992588 are located at g.60888M (NC_037334.1) and g.60834R (NC_037344.1). Therefore, the length of the bovine *CYFIP2* gene is 134,323 bp with 33 exons. The length of the bovine *SGSM1* gene is 74,039 bp with 27 exons. *CYFIP* and *SGSM1* are two immunity genes that affect the neural system [27–29]. In mice (*Mus musculus*), mutations in the *CYFIP2* and *SGSM1* genes can influence many neurological disorders [30–32].

Interestingly, the immunity genes *CYFIP2* and *SGSM1* can control the coat color patterns of Sumatran native cattle. Many coloration genes of *TYR, MITF*, and *MC1R* are also related to the immunity system of animals [33–35]. In the tropical environment, many survival-related genes such as *SUGT1, SF3A3, DSCAM*, and *HSP70* were detected as candidate economic traits in tropical cattle [36, 37]. Hence, several of the common coloration genes (*TYR, MITF*, and *MC1R*) of the animals under study may control the immunity system to adapt to tropical environments. Interestingly, the coloration gene *MC5R* was confirmed as a candidate gene that controls adaptation traits in tropical environments for East African Indicine cattle breeds [38].

In this study, many genes related to coloration in cattle were identified using STRING analysis. *TUBGCP5* and *OCA2* are genes related to *CYFIP2*. In humans, the *TUBGCP5* gene is also important for neural development [39], and the *OCA2* gene is important for pigmentation in the iris, skin, and hair [40]. Subsequently, *DOC2A* and *SLC45A2* genes are related to SGSM1. *DOC2A* plays an essential role in neurotransmitter release in presynaptic terminals [41]. The *SLC45A2* gene provides instructions for producing a protein that is located in specialized cells called melanocytes [42].

Fortunately, this investigation reported that the genotype combination (haplotype) from *CYFIP2* and *SGSM1* genes can characterize cattle with different coat color patterns. In general, the coat color of Sumatran native cattle is brown, as shown in Krui (70.00%) [2], Kaur (59.98%) [6], Aceh (90.00%) [43], Pesisir (79.82%) [44], and Kuantan (71.82%) [45] Sudrajad *et al*. [46] reported that most Indonesian native cattle (Madura, Pesisir, Aceh, Jabres, and Ongole) had genetic introgression from *Bos javanicus* and *Bos indicus* lineages. In this study, the phenotype of *B. indicus* lineage (white coat color) was observed in a few Sumatran native cattle. A black coat color in cattle is also typical of *B. javanicus* lineage, which is caused by the testosterone hormone mechanism [47]. Although this study identified key genetic markers, further research is needed to elucidate the precise mechanisms by which *CYFIP2* and *SGSM1* influence coat color. Functional studies, including gene expression analysis and gene editing experiments, can provide deeper insights into the roles of these genes. In addition, expanding the sample size and including more diverse coat color phenotypes could enhance the robustness of the findings and identify additional genetic markers associated with coat color variation.

Genetic introgression of *B. javanicus* lineage (brown coat color) may be related to survivability, adaptability, immunity, and reproduction traits. Martojo [48] supported this argument by arguing that *B. javanicus* (Bali cattle) has the genetic potential to adapt well to low-input and high-stress production systems practiced by smallholders in Indonesia.

## Conclusion

Two immunity genes, *CYFIP2* and *SGSM1*, are related to coat color patterns in Sumatran native cattle. This study revealed that the genotype combination (haplotype) in the *CYFIP2* and *SGSM1* genes can be used as genetic markers for coat color patterns in Sumatran native cattle. Further research is important to confirm these findings using a large sample of predominantly black and white coat cattle.

## Authors’ Contributions

HH and WPBP: Designed the study, collected data, performed fieldwork, supervised the study, and prepared and revised the manuscript. EH: Analyzed and interpreted the data and critically revised the manuscript for important intellectual contents. ER and JF: Collected blood samples and data of Kaur cattle. ZZ: Collected blood samples and data of Jambi cattle. YY: Collected blood samples and data of Aceh cattle. NDS and DK: Collected blood samples and data of Krui cattle. MM, DKR, SI, and AA: Collected blood samples and data of Pesisir cattle. PA, AD, and SE: Collected blood samples and data of Kuantan cattle. TA and DKR: Analyzed all samples in the genomics laboratory. All authors analyzed and interpreted the data, critically revised the manuscript for important intellectual contents, and approved the final version.
